# Delayed Aortic Stent Collapse in Blunt Traumatic Aortic Injury Repair

**DOI:** 10.1055/s-0039-3401022

**Published:** 2020-02-04

**Authors:** Abdullah Alhaizaey, Badr Aljabri, Musaad Alghamdi, Ali AlAhmari, Ahmed Abulyazied, Mohammed Asiry, Mohammed Al-Omran

**Affiliations:** 1 Division of Vascular Surgery, King Khalid University, Aseer Central Hospital, Abha, Saudi Arabia

**Keywords:** aortic stent, traumatic aorta, stent collapse, thoracic aorta

## Abstract

**Background** Endovascular stent grafting has emerged as an option to treat traumatic aorta injuries with reported significantly low mortality and morbidity. Stent collapse is one of the complications that can occur in this type of treatment. The aim of this article is to analyze the expected cause of stent collapse and to draw attention to the importance of the surveillance follow-up, as this phenomenon may occur late postdeployment.

**Methods** A retrospectively collected dataset from the two highest volume trauma centers in Saudi Arabia was analyzed between April 2007 and October 2012. A total of 66 patients received stent grafts for traumatic aortic injury and were included in the study. We apply Ishimaru's anatomical aortic arch zones and Benjamin's aortic injury grading systems. There were 35 patients with aortic injury at zone 2, 26 patients in zone 3, and 5 patients in zone 4. About 96% (63) of the injuries were grades 2 and 3, including large intimal flap or aortic wall pseudoaneurysm with change in wall contour. The technical success rate, as defined by complete exclusion of lesions without leaks, stroke, arm ischemia or stent-related complications, was 90%.

**Results** Proximal stent collapse occurred in 4.5% of patients (3 of 66 inserted stents) during follow-up of 4 to 8 years (mean, 6 years). Patients with stent collapse tended to have an acute aortic arch angle with long-intraluminal stent lip, when compared with patients with noncollapsed stents. Intraluminal lip protrusion more than 10-mm increased collapse ( *p* < 0.001). Stent-grafts sizes larger than 28 mm also demonstrated a higher collapse rate ( *p* < 0.001).

**Conclusions** The risk of stent collapse appears related to poor apposition of the stent due to severe aortic arch angulation in young patients and to large stent sizes (>28 mm). Such age groups may have more anatomical and aortic size changes during the growth. Clinical and radiological surveillance is essential in follow-up after stent-graft treatment for traumatic aortic injury.

## Introduction

For traumatic aortic injury, endovascular stent grafting has become an attractive treatment method. This is especially suitable in view of the fact that it is usually in patients with multiple trauma, most of whom have severe chest and lung injuries in addition to multiple other organs (head, abdomen, pelvis, and skeleton). Such injuries can be exacerbated if treated by open surgery. Endovascular management has become the preferred choice in such injuries, as it decreases physiological stress and reduces the recovery period. 1 2 3 4 5

Thoracic endovascular aneurysm repair (TEVAR) has been approved by the United States Food and Drug Administration for descending thoracic aneurysms. 6 As for any endovascular procedure, TEVAR has possible complications such as endoleaks, migration, paraplegia, and/or collapse. 6 7 8 However, use of the aortic stent in treatment of traumatic aortic injury seems to have unique characteristics, with unique potential complications. Of note, stent grafting for trauma is applied largely in young patients with traumatic injury and small, fragile aortas. 9

In this article, we analyze risk factors that may lead to stent collapse of the endovascular treatment of traumatic aorta injuries. We extend follow-up longer than in other literature. Young patients with traumatic aortic injury are subject to more anatomical and aortic size changes during normal growth This confers greater susceptibility to late post-stent complications and highlights the importance of post TEVAR clinical and radiological surveillance follow-up.

## Materials and Methods

### Study Design

This study is an observational, retrospective database and medical records review for all patients who underwent TEVAR for blunt traumatic aorta lesions in two trauma centers in Saudi Arabia. The data were collected during the period between April 2007 and October 2012. The study was conducted to analyze the risk factors and causes for stent collapse post TEVAR for traumatic thoracic aorta transection.

**Fig. 1:**
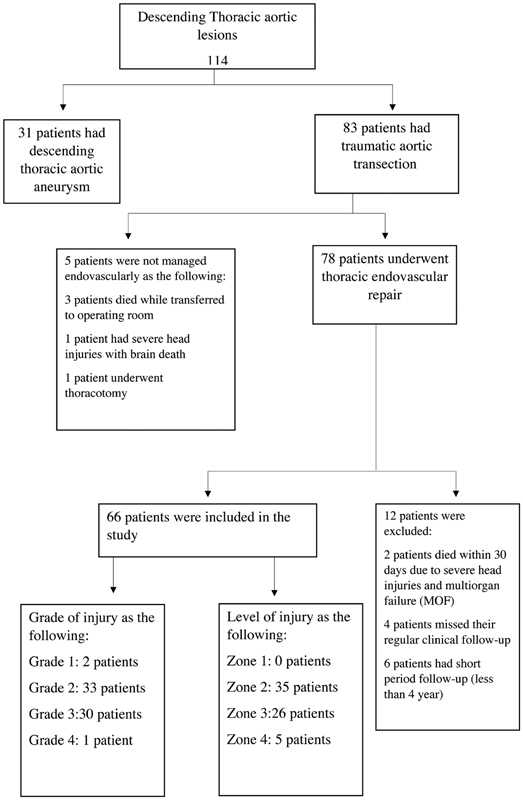
The distribution of descending thoracic aortic traumatic transection conducted in this study out of all descending thoracic aortic lesions.

We reviewed 114 patients with descending thoracic aortic lesions, 83 of whom had blunt traumatic descending thoracic aortic injuries. Sixty-six patients were managed endovascularly and included in this study, as shown in Fig. 1 .

The data were collected using a designed data collection sheet which included age, gender, time, type, and site of trauma, any other associated injury, severity of injury, anatomical distribution of aortic injury, level of injury, level of consciousness according to the Glasgow Coma Scale, operation duration, and associated intraoperative or postoperative stent complications (including stent migration, leaks, thrombosis, stroke, and/or collapse). We assessed for these complications immediately, and at 1 month, 6 and 12 months, and 2 and 5 years postoperatively.

Through this paper, we draw attention to the late sequelae for such procedures, with a special interest in young age groups. To standardize the follow-up for late sequelae in our TEVAR cases, we excluded recent TEVARS within the last 5-year period. The preoperative inclusion and exclusion flow for both centers is presented in Table 1 .

**Table 1:** Exclusion criteria for thoracic endovascular aneurysm repair in thoracic aortic injury in both centers in Saudi Arabia

Exclusion Criteria for TEVAR in thoracic aortic injury in both centers in Saudi Arabia
*Anatomical variations* :
History of left innominate artery cardiac-bypass surgery
The injuries proximal to the left common carotid artery origin; Ishimaru's zone (0 and 1)
Hypoplastic right vertebral artery
Innominate right subclavian artery
Hemodynamic instability is indication for low threshold toward open thoracotomy

Anatomical criteria included aortic arch zone, site and grade of injury's (Ishimaru's anatomical aortic arch zones and Benjamin's aortic injury grading system) 10 11 12 and measurements of aorta and stent–graft diameter, size and angles, and pre- and post-stent-graft deployments.

Computed tomography (CT) scans were reviewed to deduce the leading causes and risk factors that may result in this stent graft collapse.

Technical success was defined as complete exclusion of the aortic lesion, without conversion to thoracotomy, occurrence of Type 1 endoleak, stroke, arm ischemia, immediate stent collapse or migration, and/or mortality within 24 hours.

Institutional approval from the local ethics committee was granted for this study and patient medical records were reviewed retrospectively in the medical record department only. Patient information was kept strictly confidential. For this type of study individual formal consent was waived.

Statistical analysis was performed using SPSS program for IBM version 22. The Chi-square test and risk assessment by odds ratio and 95% confidence interval were used. *p* -Value is considered significant if less than 0.05. The characteristics for the study group including their comorbidities, pre- and postoperative complications are shown in Table 2 .

**Table 2:** General characteristics for the patients including comorbidities among the studied group

General characteristics of the studied patients		Number (%)
Mean age of the studied patients		34.7
Gender	Male	56(84.8%)
	Female	10 (15.2%)
Type of injury	Less than 10 mm from SCA	52 (78.8%)
	More than 10 mm from SCA	14 (21.2%)
Grade of injury	I	2 (3%)
	II	33 (50%)
	III	30 (45.5%)
	IV	1 (1.5%)
Other injury	Severe head injury with GCS less than 8	3
	Lung injury	5
	Abdominal injury require laparotomy	4
	Liver injury	7
	Splenic injury	5
Endoleak	I	2 (3%)
	II	3 (4.5%)
	III	0
	IV	0
Migration		0
Collapse		3 (4.5%)
Stroke		1 (1.5%)
Paraplegia		1 (1.5%)
Subclavian steal syndrome		0
Left arm claudication		0
Left arm acute ischemia		0
Respiratory complication		1
Renal failure		1
Cardiac complications		0
Groin wound complications		1
Access vessel injury		0
Conversion to open thoracotomy		0

Abbreviations: GCS, Glasgow coma scale; SCA, subclavian artery.

### Management Approach

In both trauma centers, we follow a standard management protocol for all trauma patients according to the American College of Surgeons Advanced Trauma Life Support (ATLS) management guidelines. CT chest with arterial phase contrast was done for all suspected aortic injury patients. The aorta diameter measurements were standardized in both centers and usually measured at both proximal and distal levels. We recognize the diameter variation between aortic systole and diastole (that may reach up to 17.8%) scan 6 ; we usually measured an extra two descending aorta segments distal to the lesion aiming to minimize segmental diameters variation as much as possible. We used a central line detector with 2.5-mm interval at all aorta levels (by Aquarius iNtuition CT viewer from Terrarecon). Stent sizes were chosen according to the stent manufacturer's instructions, with over sizing usually at 15 to 20%. All surgical procedures were performed in the vascular operating theater equipped with a vascular soft wire C-arm fluoroscopic machine on a carbon fiber mobile operating table.

All patients underwent intraoperative pre- and poststent deployment angiography using a pig-tail angiographic catheter through a contralateral femoral artery sheath (6 French). The stent deployment site, size, degree of over sizing, proximal and distal landing zone, site on size, and the anatomy of the aortic arch and supra-aortic arch arteries were usually reevaluated by predeployment intraoperative digital subtraction angiography.

Although there is a recent new grading system for blunt traumatic aortic injury that may provide more opportunity for conservative management, specifically for mild grade injuries, 3 10 11 13 14 we have employed the new categorization system in our management decisions only recently. During the study period, our management approach was to treat all thoracic aortic injuries using an endovascular approach aimed to avoid any future adverse sequelae. In retrospective review of all our traumatic aortic injuries, there were 2 treated patients who had minimal intimal tear, 33 patients with large intimal flaps more than 10-mm length with subintimal thrombus, 30 patients with pseudoaneurysm with abnormal aortic contour, and 1 patient with free-contrast extravasation (as per the new classification scheme for treating blunt aortic injuries, which was published by Benjamin et al 10 ).

All deployments were done during apnea mode with low blood pressure (targeting mean arterial pressure less than 80 mm Hg using labetalol infusion adjusted according to body weight and blood pressure response to help in aortic stent-graft deployment accuracy). Although lowering heart rate increases the stroke volume, which may increase the stent migration risk, on other hand the bradycardia may help in attachment of the stent to aortic wall, the stent-graft deployment mechanism takes a few seconds. Heart rate and aortic wall movement synchronization may enhance proper wall attachment and adherence to avoid stent migration, flexion, or collapse during the deployment. The proximal landing zone was 10-mm proximal to the aortic transection site. We did not have any early or delayed stent migration among all our cases. During frequent clinical follow-up, history, physical examination, and CT angiography (CTA) scan were performed for all patients. Information was gathered concerning the activity level and lifestyle changes. Blood pressure differences in the upper and lower limbs were assessed, as well as neurological changes, transient ischemic attacks, stroke, paraplegia, or quadriplegia, and radiological changes as migration, collapse, or endoleaks. Follow-up was performed at 1 and 6 months and then annually.

## Results

All clinical and procedure data for all endovascularly managed aortic injury patients are presented as in Table 2 .

There were three stent collapses. We reviewed all their pre- and poststent procedure reports. Table 3 summarizes their clinical data, time between the stent deployments and collapse, symptoms during their clinic attendance, and the improvement of their symptoms after management. Although Gore TAG stent grafts were not the only exclusive used stent grafts as it appeared in Table 4 , all our collapsed stents were Gore manufactured; two of them were the first manufactured Gore type and there was one C-TAG. This has been observed in many other stent-graft collapse publications as well. This stent-graft type has been widely used for aortic lesions internationally, having received earliest Food and Drug Administration approval. It is not clear if there is a manufacturer related risk factor, as most of international applied TEVAR stents have been Gore TAG type.

**Table 3:** Stent collapse patients summary for the presentation and treatment procedures

Patient number	Age (y)	Indication of stent	Symptoms	Time of collapse	Symptoms reliving post repair of collapse	Proximal aortic diameter (mm)	Size of first stentandoversizing	Aortic diameter at deployment site (mm)	Intraluminal lip length(mm)	CT scan	RX of collapse
1	24	Traumatic aortic dissection	Progressive persistent HTN Loss of lower limbs pulses Has no previous comorbidities	2.5 years post first stent	Immediately postrepair	24	Gore-TAG 28 × 15 Oversizing = 17%	23	15	Inner wall collapse of stent	C-Gore-TAG 31 × 10
2	40	Traumatic aortic dissection	24 hours post first stent sever retrosternal chest pain With left shoulder pain. Absent lower limbs pulses Has no previous comorbidities	24 hours post first stent	Immediately postrepair	26	Gore-TAG 28 × 10 Oversizing = 8%	24	13	Inner wall collapse of stent Type 1 endoleak	Gore-TAG 34 × 10
3	47	Traumatic aortic dissection	Shortness of breath, pulmonary edema, loss of lower limbs pulses Has no previous comorbidities	2 years post first stent	Immediately postrepair	24	Gore-C-TAG 28 × 10 Oversizing = 17%	24	11	Inner wall collapse of stent	C-Gore-TAG 34 × 10

Abbreviations: CT, computed tomography; HTN, hypertension; RX, treatment.

**Table 4:** The relationship between the stent collapse and the risk factors

	3 (+ve)	63 (−ve)	X2	*p* -Value
*Stent type* :				
Gore	3 (100%)	46 (73.01%)	1.09	0.57
Medtronic	0 (0%)	11 (17.46%)		
Cook	0 (0%)	6 (9.52%)		
*Intraluminal lip* :				
> 10 mm	3 (100%)	0 (0%)	66	< **0.001**
< 10 mm	0 (0%)	63 (100%)		
*Stent graft size* :				
=28	3 (100%)	18 (28.6%)	6.7	0.0090
< 28	0 (0%)	45 (71.4%)		
*Subclavian artery coverage* :				
yes	3 (100%)	49 (77.8%)	0.56	0.45
No	0 (0%)	14 (22.2%)		
*Over size* :				
< 10%	0	3		
10–20%	3	56	0.37	0.82
> 20%	0	4		

All collapsed stent-graft patients were symptomatic. Two patients had a late presentation, more than 2 years after stent deployment, with persistent hypertension more than 150/90 mm Hg, not responsive to triple-drug pharmacologic therapy for 3 months before the clinic visit. The third patient presented via the emergency department with acute, sudden abdominal, and bilateral lower limb pain, 1 month after stent deployment. All the patient's symptoms were relieved immediately after the treatment of the stent collapse as summarized in Table 3 .

We expected that multiple factors could lead to a collapse of the stent (proximal aortic diameter, size of stent, degree of oversizing, aortic-arch angulation, and intraluminal lip stent length). However, there was no significant oversizing or under sizing in all collapsed stents (18, 17, and 17%) as the proximal aortic diameters were 23, 24, and 24 mm with stent grafts applied of size 28 mm × 15 mm, 28 mm × 10 mm, and 28 mm × 10 mm Gore-TAG. The intraluminal stent lip protrusion length was 15, 13, and 11 mm in all collapsed stents.

**Fig. 2:**
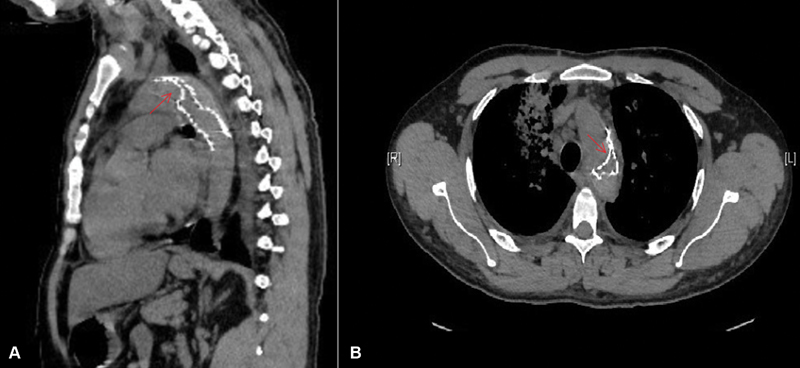
Computed tomography for chest showed inner (medial) wall collapse of the stent graft inserted in traumatic aortic transection as showed in arrow in sagittal view ( **A** ), and ( **B** ) showed axial view in collapsed stent graft as showed in arrow.

Our data revealed that intraluminal lip protrusion of more than 10 mm led to a high risk for collapse ( *p* < 0.001; Figs. 1 and 2 ). An increase in the intraluminal lip protrusion inside the aortic arch more than 10 mm led to a significant risk for collapse ( *p* < 0.001) possibly due to lack of devise/wall apposition and exposure to high blood flow ( Figs. 1 and 2 ). A stent graft size larger than 28 mm also showed a higher collapse rate ( *p* < 0.001) as presented in Table 4 .

**Fig. 3:**
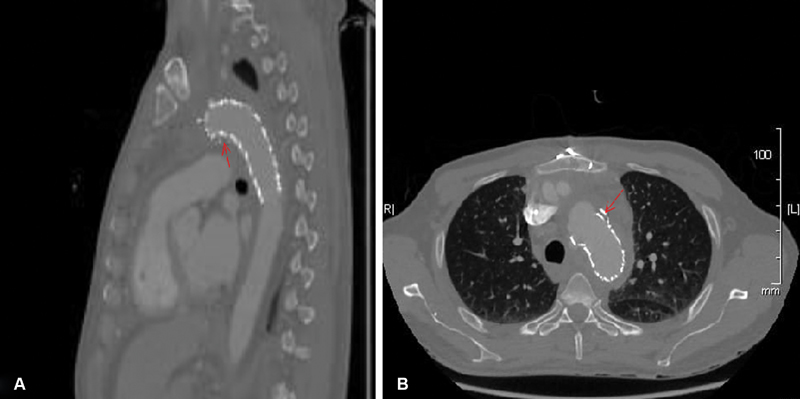
Computed tomography for chest showed postcollapsed stent graft endovascular treatment by reinsertion for other stent graft and ballooning as showed in the arrow in sagital view ( **A** ) and also showed in arrow in axial view ( **B** ).

Treatment of these stent collapses was approached using an additional endovascular stent in the proximal landing zone. We used C-Gore-TAG devices (31, 34, and 34 × 10 cm). We founded it difficult in collapsed stent to emerge the second stent graft proximal to the primary stent without ballooning to open the collapsed part and we usually used to reballoon the second deployed stent postdeployment for maximum sealing with the primary stent to avoid leaks between the two stent grafts that may lead to father complications and there was no leak or recoil and no complication intraoperatively and postoperatively ( Fig. 3 ).

Among 66 endovascular stents used for traumatic aortic transection, 52 (78.8%) intentionally covered the left subclavian artery, as all the traumatic transections were at the aortic isthmus within 1 cm from the left subclavian artery. In all cases of collapsed stents, the first stents applied covered the left subclavian artery during treatment of the aortic injury. The third patient had the first stent deployed, during the treatment of the acute aortic traumatic lesion, just at the distal edge of the left common carotid artery (LCCA). It was a difficult decision, at the late corrective surgery, to expose him to a surgical procedure or even a hybrid surgical bypass of the LCCA before stenting, as the patient was an active young man. The giant stent (or Palmaz stent) is a treatment option but because of the acute angulated aortic arch and friable aorta, we preferred for this patient the C-Gore-TAG which has a 5-mm bare metallic proximal edge and more radial force. This was deployed successfully with the bare metallic part just at the origin of the LCCA. Angiography showed a patent LCCA. Postoperation, the patient was fully conscious with no neurological or vascular deficit. He was discharged in good clinical condition.

All patients underwent follow-up CTA within 1 month after endovascular treatment. All stents were patent and no complications were detected. All the patients continue with regular annual follow-up CTA.

## Discussion

**Fig. 4:**
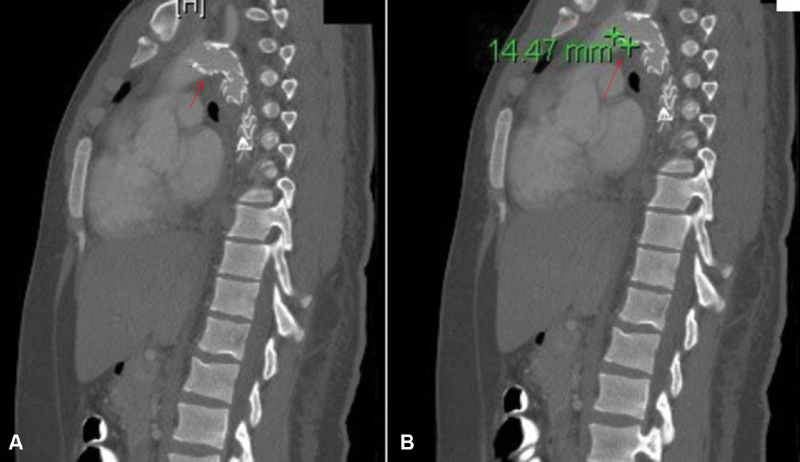
Sagital view for chest computed tomography shows inner (medial) lip for the stent graft that increased the risk for collapse as shown in arrow ( **A** ), while arrow in ( **B** ) shows stent inner lip length more than 10 mm in high angulated traumatic aortic arch.

Most traumatic aortic lesions are at the left subclavian artery level near to aortic isthmus at the ligmentum arteriosum attachment with medial edge of aortic wall that is beginning of acute aortic arch in most of young-age patients group and due to acute aortic arch angle in young-age group associated with proximal injury site near to aortic arch, the proper stent deployment landing zone 20 mm (>10 mm) lead to stent-graft protrusion inside the aortic arch lumen. Muhs and associates 9 reported several factors that may lead to stent collapse including acute aortic arch angulation and poor apposition of the stent graft to the aortic inner curvature. An increase in the intraluminal lip protrusion inside the aortic arch more than 10mm led to a significant risk for collapse ( *p* < 0.001; Fig. 4 ). Endovascular stent grafting is a recommended method of treatment for traumatic aortic injury, 5 and in our experience with 66 patients who were treated for traumatic thoracic aortic injury, we found the endovascular stent graft to be safe and minimally invasive with low mortality and morbidity rates.

The endovascular stent-graft procedure has its own advantages and disadvantages as do other surgical procedures, and stent collapse is one of the complications in the management of such aortic injuries.

Following any endovascular stent-grafts procedure, surveillance is recommended. Annual and radiological “contrast CT” is still the common follow-up approach post-endovascular aortic repair for abdominal aortic aneurysm for most authors. 15 16

TEVAR for management for traumatic aortic injury has no generally agreed radiological follow-up approach.

Postoperatively, endoleaks, stent-graft migration, and stent collapse are the common possible complications identified by most of authors. Canaud et al 6 reported a critical high-risk period for aortic stent collapse in the first month after implantation. However, Rodd et al 17 reported that two of three stent collapses occurred between 6 and 11 months after implantation. In our experience, two of three collapsed stents occurred at a longer time after implantation (2 years and 2.5 years after implantation).

Although we reported that patients with collapsed stents were symptomatic at presentation, there are multiple patients in many published papers who were asymptomatic. 7

Because stent procedures for traumatic aortic transection are applied in young age groups, annual CTA may expose them to considerable radiation burden. Till now, there is no consensus posttraumatic aortic injury TEVAR surveillance protocol. One month and 1-year post-TEVAR clinical and radiological check will likely disclose most complications. Then, each 5 years may suffice.

Muhs et al 9 reported multiple suspect anatomical factors associated with acute endograft collapse after Gore-TAG treatment of thoracic aortic dissection and traumatic rupture. These include the proximal aortic diameter, distal aortic diameter, intraluminal lip length, arch curvature, lip-to-arch angle, completeness of stent graft apposition, percentage of over sizing, and smallest aortic diameter. 6 9

In our retrospective review, the collapsed stent-graft patients have acute aortic arch angles, leading to stent-graft protrusion into the aortic arch lumen. An increase in the intraluminal lip to more than 10 mm led to a high risk for collapse. Also, lack of device wall apposition will expose the device to high blood flow energy that may contribute to stent collapse. On other hand, stent characteristics play a role in such complications, as well as stent graft sizes larger than 28 mm, also demonstrated a higher collapse rate. Cabrera highlights the effect of stent's diameter on radial and hoop forces, noting significant decrease in radial forces for stents with diameters larger than 26 mm. 18

In other view, due to aorta tissue friability in acute traumatic aortic transection and small aorta size, most vascular surgeons leaning towered the conservative side in oversizing 15 to 20% oversizing and avoid the oversizing over 20% that may increase the migration of the stent graft. This is not a significant factor for the collapse as showed with us, as well other retrospective studies. 7 12 14 15 19

Through the above-mentioned findings, we would like to draw attention for the importance of both the patient's anatomical varieties and the stent's mechanical factors in such complications. Endovascular aortic stent collapse may be managed with different methods, including using a Palmaz giant stent, proximally extending grafts using other stents and even open surgical repair using cardiopulmonary bypass. 6 7 11 13 14 17 We prefer the addition of another stent proximally after balloon angioplasty of the collapsed stent in same procedure.

Balloon angioplasty of a collapsed stent alone is an insufficient method to treat this complication. If necessary, the collapsed stent can be removed either later or at the same time. 14 19 Improvements in endovascular stents may help in the prevention and treatment of endovascular complications. The C-Gore-TAG devices enable endovascular management of such complications, especially in more complicated cases in which the original deployment is just at the left CCA origin, in which the proximal 5-mm bare metallic portion can safely be used at the origin of the LCCA.

## Conclusions

The endovascular management of traumatic aortic injury is safe, minimally invasive, and commonly applied in patients with critical multiple-organ injury. Anatomical characteristics and stents' mechanical factors play a role in stent-graft collapse. The risk of stent collapse is related to poor opposition of the stent due to severe aortic arch angulation in young patients. Protrusion of the stent in to the aortic arch lumen (increasing the intraluminal stent lip length) also contributes; an intraluminal lip of more than 10 increases risk of collapse. Young aortic transection patients are subject to more anatomical and aortic size changes, as they grow older. Clinical and radiological surveillance are essential.
